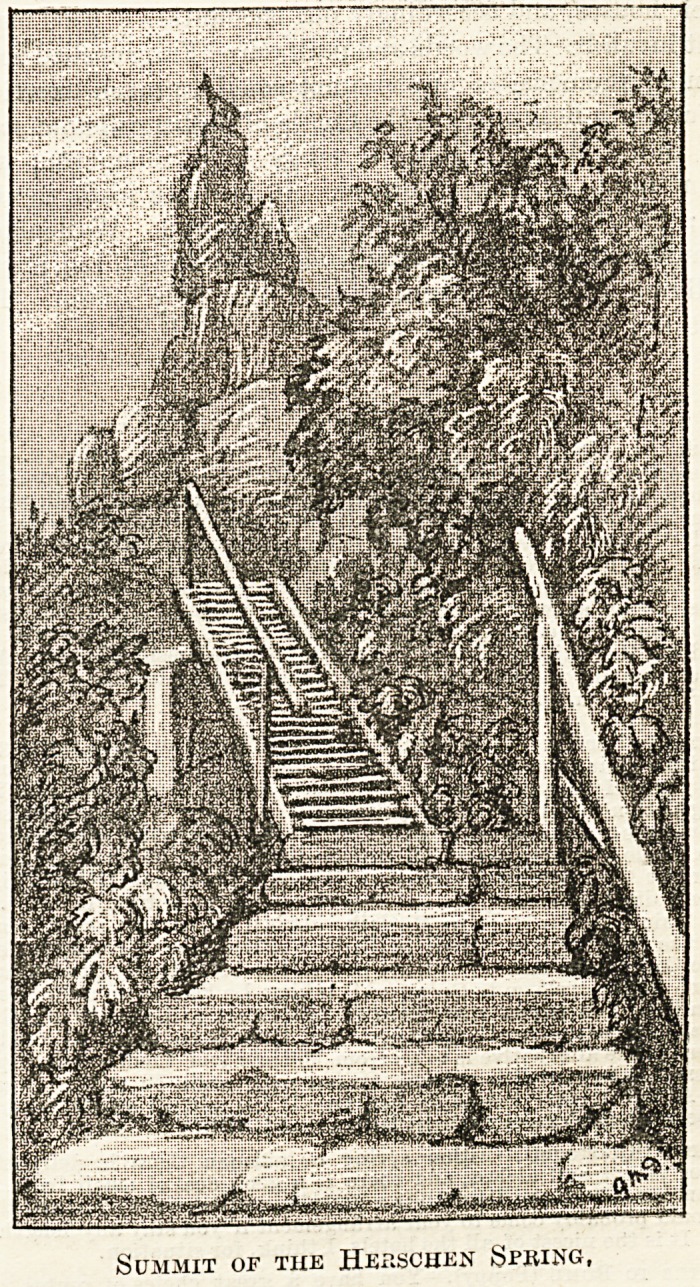# "The Hospital" Nursing Mirror

**Published:** 1899-05-20

**Authors:** 


### "The Hospital" Nursing Mirror.

Being the Nursing Section of "The Hospital."

[Contributions for this Section of "The Hospital" should be addressed to the Editor, The Hospital, 28 & 29, Southampton Street, Strand, London, W.C., and should have the word "Nursing" plainly written in left-hand top corner of the envelope.]

### Notes on News from the Nursing World.

### MISS FLORENCE NIGHTINGALE.

It is with the greatest satisfaction that we are assured the health of Miss Florence Nightingale gives no particular cause for anxiety, and we are sorry that we should have given wider circulation to a report which was current last week. We know it will afford the utmost pleasure to our readers to learn that Miss Nightingale's medical attendant considers that she is in her usual state of health, which has, in fact, undergone no special change of late.

### THE CAFE CHANTANT AT THE HOTEL CECIL.

The entertainments in aid of the Royal British Nurses' Association formed quite the social event on Monday. Complete and brilliant success crowned the efforts of Mr. and Mrs. George Alexander. The visitors during the day included the Princess Christian (president of the association), the Prince of Wales, Princess Henry of Battenberg, the Duchess of York, the Duchess of Connaught, and the Princess Victoria of Schleswig-Holstein, as well as nearly all the patronesses. As to the programme, with the "extras," more than a hundred items were submitted by the ladies and gentlemen of the musical and dramatic professions, who gave their services on the occasion. The Grand Hall and the Victoria Hall were crowded both in the afternoon and evening. Superbly decorated, with a brave show of beauty and fashion, they presented a very striking appearance; and the absence of formality in the arrangement of accommodation for the audience added to the picturesque character of the gay scene. If we forbear from mentioning by name any of those who contributed to the enjoyment of the proceedings, it is only because the whole of the wonderful programme was carried out in the most satisfactory manner.

### MR. CHARLES WYNDHAM ON NURSES.

Everybody regretted the absence through indisposition of Sir Henry Irving from the Café Chantant. But Mr. Wyndham, in his opening speech in the Victoria Hall, made some singularly happy remarks. The very title of the Royal British Nurses' Association, he said, fell upon the ear like a chime of bells. "Each of these three names, Royal British Nurses, which for more reasons than one might be called 'Christian' names, stirred the deepest chords of our nature. The first appealed to our loyalty, the second to our patriotism, and the third to our humanity." For, after all, continued Mr. Wyndham, "which of the Seven Stages of Man is exempt from the necessity of a nurse's care? She lifts us into the cradle when we enter upon life; she ministers to us in our suffering in our journey through; and at the end, by her tender services, smooths our path towards the Silent Land." Having paid this moving tribute to the members of the nursing profession, Mr. Wyndham alluded to the strong support and countenance given by the Royal Family to the association, and to the fostering care of Princess Christian, "who for twelve years has taken such keen and loving interest in its prosperity." He concluded by asking, "Who, under the circumstances, could refuse to lay at the feet of the nurses the talents, whether of gold or brain?" Certainly there was no refusal on Monday. The talents of brain were lavishly offered to the association, and, as the result of an enquiry, we learn from Mr. Alexander that the profits are roughly estimated at £1,500, "more or less."

### THE SALARIES OF NURSES IN THE TROPICS.

The appeal of the Colonial Nursing Association for funds has provoked from a correspondent of the *Standard* an intimation that the salaries offered to nurses in the tropics are often utterly inadequate. This is a matter of vital importance. Of all nurses those who work under the most wearing and frequently the most perilous conditions ought to be sure of earning what is called a living wage. The case is mentioned of a hospital on the Equator where a matron is paid 100 rupees a month, without rations or allowances, and the assistant matron 60 rupees a month with 15 rupees for allowances, and no rations. The hospital contains between 30 and 40 beds, and the ladies in charge are not allowed the assistance of a coolie to sweep the room. The cost of the outfit for the voyage out and for use on arrival is not less than £25. The Colonial Nursing Association, we are sure, will recognise that arrangements of a very different kind from these must be made in order to secure for the colonies the requisite talent and devotion.

### A DANGER TO BE AVOIDED BY DISTRICT NURSING ASSOCIATIONS.

It is one thing to advocate the appointment of a trained nurse in every village that is large enough to possess a parish council and quite another to support the policy of allowing nurses to attend cases without a doctor. Mr. Hanbury Frere, M.B., of Stradbroke, Eye, writes us that, "as some district nursing associations have permitted their nurses to attend cases without a doctor, trusting that if anything went wrong the doctor would take over all responsibility, he has ventured to put forward certain difficulties into which a nursing committee are likely to get if they pursue this course." He says that "the idea seems to be prevalent that if the nurse has the L.O.S. she can take cases without the doctor," and he encloses a copy of a pamphlet which he has written upon the subject. There are many useful suggestions in it, but the most practical for the avoidance of the difficulties to which Mr. Frere refers in detail, is that it should be made a rule by the committee of a nursing association to include the local practitioners among the members, and that the nurse should be sent only at the request, or with the consent, of the medical attendant. Mr. Frere maintains that it is to their own interest that the committee should make such a rule, since, in case of an inquest, any blame would attach to them rather than to the nurse; and he asserts that such an occurrence as public censure would in all probability "destroy all chance of working a nursing scheme in that district, at any rate for many years to come."

### THE MATRON AND NURSES AT SCARBOROUGH WORKHOUSE INFIRMARY.

The final result of the investigations by the Scarborough Board of Guardians is that Sir Charles Legard and his colleagues declare the charges brought against the matron by the four infirmary nurses to be "wholly disproved"; and, in these circumstances, it was decided to recommend the acceptance of the resignations of the nurses. It may be remembered that the investigation was demanded by the superintendent nurse, Miss Barralette, and supported by the other members of the nursing staff, Miss Flynn, Miss Sinclair, and Miss Clark, who threatened resignation unless it was conceded. Some of the Guardians opposed the inquiry, but the chairman of the Hospital Committee and others were in favour of it. The accusation against the matron was that while, in the interests of the patients, she urged the superintendent nurse to apply for an additional nurse, she stated elsewhere that no addition to the staff was needed, and it was also asserted that she was profoundly ignorant of the diseases and treatment of the patients. This is one more illustration of the difficulties which are apt to arise in workhouse administration in consequence of the subordinating of the nurses to the authority of the matron in regard to every minute detail of ward management.

### PATIENTS WHO WON'T GO INTO HOSPITAL.

Nurses who are likely to have to work in Natal will be interested in the following from a correspondent at Durban: "It takes very little to upset our native servants, and they leave us and go home on the slightest provocation, without considering the inconvenience they may cause. The fact that they ultimately nearly always return does not always compensate for present discomfort. Just now we have a miserable substitute for our driver (coachman), who left us because his child was ill, apparently from paralysis. I tried to persuade him to bring her to the hospital here, where natives are received free of charge, and where now there is an excellent doctor and a reliable staff of nurses. He said that if he found the illness of the kind an English doctor could treat he would do so; but if the disease had been put into the child by an evil-minded native, only a native doctor could cure her. As he would consult the native first. the verdict, of course, was a foregone conclusion. The belief in witchcraft among the Kaffirs remains as strong as ever, notwithstanding all teaching to the contrary."

### NURSES IN COUNCIL.

The discussions on some nursing topic which take place monthly at the Trained Nurses' Club, 12, Buckingham Street, Strand, are always much appreciated by members and others who have the opportunity of being present on these occasions. Many subjects have been considered, and the debates are very helpful. "Rational Dress for Infants" is the subject for discussion this month, and the debate takes place at the club on Friday evening at eight o'clock. On this occasion it is an "open evening" for members and their friends, and a good attendance is hoped for. There will be a display of baby garments of various sorts and kinds. How best to clothe babies is a question of interest to all nurses, especially midwives and monthly nurses, and a comparison of views and experience is certain to be instructive.

### LONDON SCHOOL NURSES' SOCIETY.

The annual meeting of the London School Nurses' Society will be held on June 19th, when the chair will be taken by Lady Aberdeen. Lord Reay and Mr. Brudenell Carter are amongst those who have promised to speak. The work of this admirable society is gradually extending. Four nurses are now employed in Hackney, Tower Hamlets, Southwark, and Greenwich, some 37 schools in all being visited. Excellent results are found to follow their ministrations; in fact, it is stated that "bad eyes and dirty heads are practically stamped out of a school by six months' regular visiting, and that consequently each nurse is able to enlarge the scope of her work." The Queen Victoria Jubilee Institute has set the seal of its approval on the society, and inspects their nurses. Wherever it is possible the work will be done by a Queen's nurse. The committee urgently appeal for subscriptions and donations to enable them to carry on their work, and especially provide them with funds to start three more nurses at once in the districts where they are most needed. Subscriptions should be sent to the hon. treasurer, W. C. Bridgeman, Esq., 89, Harley Street, W.

### SHORT ITEMS.

At a general meeting of the subscribers to the Westminster Queen's Reign Commemoration Fund in the Town Hall on Monday a draft deed was submitted by the solicitor to the vestry directing that the income of the capital fund created should be paid in aid of the nursing institute which has already been founded in Westminster, and that the trustees recently appointed, namely, the Duke of Westminster, Sir J. Wolfe Barry, Mr. Burdett-Coutts, M.P., Mr. F. K. Rose, and Colonel Davies Sewell, should be requested to sign the deed accordingly.—On the afternoon of Friday next week an entertainment will be given, under the auspices of Lady Cottenham, in Lady Bute's ball-room, for the benefit of Roman Catholic district nurses who work unpaid among the poor. The entertainment will consist of the performance of a little French comedy and music.—Over £850 was realised at the bazaar held last week at the Southend Pier Pavilion in aid of the Southend Victoria Hospital and District Nursing Association.—Under the will of the late Mr. Vincent Stuckey Lean several of the hospitals benefit. Mr. Lean has left £1,000 each to King's College Hospital, Bristol General Hospital, the Bristol Hospital for Sick Children, and the Sanatorium at Weston-super-Mare, as well as £20,000 to the directors of the new orphan homes on Ashley Down.—Last week the Queen conferred the decoration of the Royal Red Cross upon Miss Theresa McGrath (who was specially attached to the hospital of the 36th Sikhs at Gulistan), Miss Catherine Sarah Mowbray (late of the Indian Nursing Service), and Miss Sarah Lucy Wilshaw (Army Nursing Service) for special devotion and competency in nursing the sick and wounded at Gulistan, Malakand, and Egypt, during the recent campaign in the Soudan, respectively.—The Rev. W. H. R. Longhurst has been presented with a library chair and an illuminated address by the members of the nursing staff, on the occasion of his resignation of the office of chairman of the Executive Committee of the Worcester Infirmary. The address was signed by the matron and 43 nurses past and present.

### On the Modes of Dying and the Signs of Death.

(*Concluded from page* 86.)

### SIGNS OF DEATH.

The following is the conclusion of the lecture by T. E. Hayward, M.B., F.R.C.S., of which we gave some portions in our last issue :—

"When a patient has finally ceased to breathe, and his heart has ceased to beat, death has occurred. That this is so is usually obvious even to an ordinary looker-on without any special experience or knowledge. The "death-like pallor," the indescribable but very definite aspect, the absolute stillness, ere long followed by the icy coldness of the corpse, then by the stiffening of the limbs, in the vast majority of cases leave no room for doubt. And yet, though very rarely, cases do occur in which the most skilled and trained observer is at a loss for a time to definitely pronounce an opinion as to whether the patient be really dead or not. It should be known to you all that some persons have a life long horror of being buried alive, and sufficient instances have been well authenticated of this too horrible event to make it desirable that it should be at least considered as a bare possibility.

"You may hereafter sometimes be placed in circumstances in which you may have, for the sake of the patient's friends, and to give a definite and well-founded opinion that the patient whom you and they have been watching over is really dead and has passed the barrier through which there is no return. It is therefore desirable that you should know how to proceed in making such a systematic examination of the supposed corpse as shall convince you that the 'river of death' has really been crossed. To do this you have to recall to mind that the two essentially vital functions are those of circulation and respiration.

"The examination, then, of the body of a person supposed to have recently died resolves itself into an investigation directed to ascertain whether there are discoverable any signs of the heart's action or of the movements of breathing. Seeing that, as has already been said, breathing may have ceased for a time while the heart still goes on beating, it is obvious that the temporary cessation of breathing does not prove death; on the other hand, if the heart has absolutely stopped for a very limited time, at the very outside for a few minutes, death is certain. It is evident, therefore, that you should first endeavour to find out if any sign exists of the continuance of the circulation of the blood, and you would proceed thus :—

"1. Feel the pulse at the wrist. If the feeblest flickering pulsation is perceptible you will know that life is not yet extinct; but failing to feel the pulse is by no means sufficient proof that the heart is not acting.

"2. Let the hand next be placed over the region of the heart to discover if you can feel any beating there; you may thus become sensible of such feeble action as will be quite insufficient to give a pulse at the wrist.

"3. If you fail to feel anything, try if you can hear any heart-sounds—either by applying the ear directly or by means of the stethoscope. To be sure that no sounds exist you may have to listen for a minute or two with the most perfect surrounding stillness.

"4. It is possible, however, for such excessively feeble action of the heart to be continuing as to give no evidence either to feeling or hearing. The next test, then, which may be employed is to tie a piece of string or tape rather tightly round one of the fingers. If circulation be going on, however feebly, in a little while the part of the finger furthest away from the ligature will become discoloured and swollen.

"If these tests give negative results it may be concluded with almost absolute certainty that the heart's action has ceased, and, therefore, that death has taken place. Still, as in doubtful cases, it is desirable to get all the evidence obtainable, and not to depend on one or two signs alone, you should next use the three following tests, simple, but of a high degree of delicacy, to find out whether any such feeble attempts at breathing are going on as are imperceptible to ordinary observation :—

"1. The 'mirror test'; that is, holding a small looking-glass in front of the nose and mouth to see if any breath moisture is deposited upon it.

"2. Placing a feather or a little fleck of cotton wool on the upper lip to see if it is moved, as it would be by the faintest breath.

"3. Place a saucer, partly filled with water, on the chest, and put a lamp with a steady flame where its reflection from the surface of the water will be thrown on the wall or ceiling. The slightest possible movement of the chest will be thus rendered visible, magnified by the oscillation of the long index of the beam of light.

"If these signs, too, are all negative, it may be concluded almost beyond doubt that the two vital functions have ceased and that the patient is therefore dead.

"At this point the question may be asked, What are the cases in which any doubt may exist for a time as to whether death has really occurred or not? There are three conditions in which death may be apparent, but not real: (1) Syncope, (2) Asphyxia, (3) Trance.

"1. Syncope.—This word has already been used to denote sudden death from complete stoppage of the heart, but the word is also used to mean sudden partial failure of the heart's action, and the cases of syncope now being considered are those in which a prolonged death-like fainting fit exists. In such cases some evident signs of life will be almost certainly made out by the systematic adoption of the mode of examination just described, and the patient may very probably recover on means of resuscitation being used, such as the access of plenty of cool fresh air, dashing cold water over the face, holding spirit of ammonia over the nose, and the administration of stimulants such as the hypodermic injection of ether, or the giving of an enema of brandy, the patient being placed meanwhile in the recumbent posture with the head rather lower than the body.

"2. Asphyxia.—In this condition no sign of breathing exists, but the heart will be found to be beating, though faintly, and attempts at resuscitation by artificial respiration may be successful.

"3. Trance.—There are some exceedingly rare cases of what is called 'prolonged death-like trance,' in which the patient lies absolutely motionless, with death-like pallor and cold surface, with no evident signs of circulation or respiration to be discovered even after using all the tests which have been described. These cases may be compared in some degree to that condition of partly-suspended and feebly-existing vitality in which some species of animals pass the winter season, and which is known as hibernation, and it is in such cases, more than all others, in which the bare possibility already alluded to of being buried alive exists.

"The examination then of a patient supposed to be dead has two practical points associated with it: (1) To discover if any hope of resuscitation exists; (2) to guard against the chance of being buried alive. The system of examination already described would have reference chiefly to the first of these points. In order to deal with the second, and guard against the possibility suggested, we have now to consider those signs of death which require time for their development, and which are so absolutely certain as to leave no room for doubt:—

"1. LOSS OF HEAT OR COOLING OF THE BODY.—When death occurs (except in some very rare cases, in which, for a time, the body temperature rises) the body begins at once to cool. This is soon apparent on the surface of the skin, indeed, in many cases it has begun to be perceptible before death. The cooling of the internal parts is, of course, brought about more slowly. In any doubtful case the temperature may be taken at intervals in the rectum. If though considerably below normal, it should remain for 24 hours constant, a presumption may be raised that life still exists, but if it were found to be gradually sinking lower and lower, it would be a certain sign of death.

"2. LOSS OF THE POWER IN THE MUSCLES OF CONTRACTING UNDER THE STIMULUS OF ELECTRICITY.—This is a test which, as a rule, only the medical attendant would apply. But for the sake of completeness it may be incidentally mentioned here, although the body, as a whole, may be dead, some of its separate parts such as the muscles may for a time retain their individual vitality. For a little time after death it will be found that the muscles "react" to the application of electricity. But this certainly ceases to be the case after three hours. Therefore, if three hours after apparent death it be found that no evidence of muscular contractility be obtainable, this may be set down as a certain sign of death.

"3. POST-MORTEM LIVIDITY.—When the body is dead the blood tends to sink by the influence of gravitation, to the lowest parts of it, and in about eight hours it will be found that on the back there are patches of livid discoloration, which go on increasing in size.

"4. RIGOR MORTIS.—This is the name given to that stiffening of the trunk and limbs which comes on after death through a kind of coagulation of the juices of the muscles. It varies very much in the time after death at which it comes on. It may occur almost immediately, or it may be deferred for many hours. The longer deferred in its onset the longer it lasts, and the earlier it begins the shorter time does it continue. When it does appear, and it almost invariably does so within twenty-four hours, it is a certain sign of death.

"5. The last sign to be noticed, and one which is absolutely conclusive, is putrefaction—that is commencing decay of the body. This usually makes itself first evident not later than in the second day by a greenish discolouration of the abdomen.

"You may thus gather that, with proper care and attentive examination, the chances of being buried alive are infinitely small.

"Should it ever happen to any one of you to see the one case out of a million in which, after apparent death for forty-eight hours, there is a constant temperature in the rectum, a continuance of electrical contractility in the muscles, no post-mortem lividity, no rigor mortis, and no sign of putrefaction, you may have ground for suspecting that you have to do with a real case of trance, but even then the responsibility of pronouncing an opinion will not be yours, but that of the medical attendant."

### Typical Patients.

### THE PHILOSOPHIC PATIENT.

HE was a day labourer, and with the ill-fortune which apparently attended him through his life (finely exemplified by his choice of a wife) by some little carelessness he allowed a heavy beam from a cart which he was loading to slip and strike his knee. It did not seem a serious injury, but continued dull pain compelled him tardily to show his knee to the dispensary doctor, with the result that he became an inmate of my ward. His philosophic temperament early displayed itself. By no means backward in asking for things, such as beer, rashers, eggs, &c., he never seemed in the least surprised or put out by a refusal. When I endeavoured to soften the harshness of the denials by reminding him that he was only one of forty, and that if he were permitted luxuries how could they be refused to the remaining thirty-nine, he would reply with the sweet, patient smile which endeared the rough, ignorant man to us all, "In coorse, Sister. I didn't mind o' that when I spoke; but I am beholden to you for what I gets, and it's all good—all good," he repeated emphatically.

My night nurse had been very good to him, and he was fond of her. She was leaving to be married, and we thought that it would be a blow to him, but by no means so. Philosophy rose to the occasion. One of the probationers, vexed with such apparent indifference, reproached him.

"But are you not sorry, Jones? You always liked her so much, and she has been very good to you."

"Aye, aye," he answered meditatively; "she has been very good, very good. One goes and another comes; I shan't be left, tho' I ain't denying as she has been uncommon kind."

A short silence followed whilst the sluggish brain was evolving an unpleasant thought. "If sister was to go now we might kick up a dust, for" (glancing round on the staff nurse and two probationers, all assiduously ministering to his wants) "she is a more 'header' nurse than any on you!"

As time went on he did not improve, and his leg was amputated. At first naturally he grieved, but by the end of the week he had found a silver lining to the cloud.

"You see, Sister, just at first I'll be forced to trust to the parish for help with the missus and the children, but after a bit, when I gets my wooden Johnny, I can nip round the villages selling things as everyone must have—tea, candles, tobacco, and such like, and most everyone will be ready to help a one-legged chap."

One day, whilst enjoying one of those chats which I always felt did me good, he unfolded a brilliant idea for the future.

"Seems to me, Sister, as if some day I might lay by a bit, little by little, and buy a donkey."

You should have seen him turn his eyes on me to ascertain if I were suitably impressed by the magnificence of the conception.

I expressed unbounded admiration for the bold enterprise, and he continued complacently,

"My grandfather—he had only one leg, lost it on the line—'tis foolishness risking your life in them flyaway things—leastways, if you can help it—anyhow he had but one leg, and he bought a donkey and a shay, as purty a turnout as anyone could see. To be sure (with a slight sigh) he were a bachelor man."

His thoughts probably, in spite of his loyalty, reverted to the presiding genius of his ill-kept home. After a momentary pause he continued—

"But then He as sends the children mostly sends bread for 'em to eat, somehow."

Alas, poor Jones! his golden visions of a donkey and "shay" and a wooden Johnny were never realised; his life steadily ebbed away. Always of a poor constitution, hard work, insufficient food, and exposure to all the evils of wet clothes, with no comfort at home, he was too weak to struggle round, though I think he lived three months after the operation.

We all missed and regretted him, and I often thought that his philosophy was really the outcome of a simple faith, unknown perhaps to himself, that surely the Lord of all the earth must do well.

### Nursing in New Zealand Hospitals.

### A CHAT WITH MRS. GRACE NEILL.

Taking advantage of the presence in London of the Deputy Inspector of Lunatic Asylums in New Zealand, who is also a lady well known to be thoroughly acquainted with all hospital work, I asked Mrs. Grace Neill to spare me a short time to tell me, as far as possible, the position of nursing in the Colony. My first question, however, was of a personal character, and had reference to her remarkable official status.

"Is it a usual thing in New Zealand for a woman to occupy the position of assistant inspector of asylums?"

"Not at all. I am the only woman who has received such an appointment, not only in New Zealand, but, as far as I know, throughout the whole of Australasia. Pioneer work seems to have been rather in my line, for when I was in Queensland I was the first woman to serve on a Royal Commission."

"How many inspectors are there?"

"Two, the Inspector-General (Dr. Duncan McGregor) and myself, and we work together. All the asylums, of which there are seven in the island, have to be visited by one of us every two or three months, sometimes more frequently."

"Have you to inspect any other institutions than lunatic asylums?"

"Oh, yes; the duties of my post include the inspection of all hospitals for the sick as well as for the insane, and also of all charitable institutions. But the hospitals are visited less often, generally once a year."

"Had you any hospital experience before you left England?"

"Early in the seventies I was trained at King's College and Charing Cross. Then for two years I held the post of Superintendent of the Children's Hospital at Pendlebury near Manchester, until I married. In 1890 I took up journalism in Queensland, but I found the climate too trying, and three years later I went to New Zealand, and was first inspector of factories, and four years ago received my present appointment."

"You have had plenty of experience of life, Mrs. Neill, some of which must be very valuable in your present position. Now as to the hospitals in New Zealand. What is the number?"

"We have only four which have over 100 beds; they are at Auckland, Wellington, Christchurch, and Dunedin. But there are between 40 and 50 smaller ones for local districts. All the hospitals are under the control of Hospital Boards, but the asylums are managed directly by the State."

"How are the hospitals supported?"

"Principally out of the rates. In old times the Government used to vote a grant when required, but this was not satisfactory, as, owing to influence, sometimes one hospital got a larger grant, whilst another, which was equally deserving, had to be content with a smaller one. Accordingly, in 1885 matters were placed on a more substantial footing by Act of Parliament, and the rates levied by district boards are subsidised by the Government. The assistance given by the Government is a substantial one. To every £1 collected in rates the Government adds another £1; to every £1 given in voluntary contributions it adds £1 4s."

"Does the system work satisfactorily?"

"Not exactly. There are many disadvantages, but in a new country like New Zealand it seemed the only course open."

"Have you any difficulty in getting nurses?"

"Not the slightest. There are always more girls waiting their chance of becoming probationers than openings provided for them. Many are glad to commence as 'emergency nurses.' They come into the hospital for the sake of the training and wait their chance. The probationers train for three years, as in England, but the salaries run higher."

"Much higher?"

"They commence at about £25, and those for nurses go up to £50. Matrons earn from £100 to £150, though in some of the smaller hospitals they may possibly not exceed £80."

"What about dress?"

"The indoor uniform varies according to the locality, but it is very unusual to wear an outdoor one, and only two or three hospitals have a regulation cloak and bonnet."

"Are the hours as long as in some of our large institutions?"

"No, because in hospitals, as elsewhere in New Zealand, there is constant endeavour to reduce hours of labour, and in most hospitals the nurses' hours are eight or nine. The shorter periods of consecutive work are decidedly a boon to the nurses, but I am not so sure about the benefit to the patients, though it is naturally a many-sided question."

"I suppose that in your official position you have to inquire into this question. Do you find the fact of your being a woman helps or hinders you in your inspection?"

"I find that the matrons and nurses are glad to have a woman, who can help with advice in certain details difficult to talk over with a man. Having the Woman's Franchise in New Zealand brings women more to the front than in a country where they are not allowed to vote."

"Do the natives, the Maories, assist at all in the rough work of the hospital?"

"Oh, no. It is always difficult to make the English people understand that the Maories are really the aristocracy of New Zealand. They are the owners of the land, and the English pay them rent. They never work if they can possibly help it. At present an experiment is being made in one of the hospitals, where two Maori girls have entered as probationers. At the end of their three years' training they will return to their own people, and, should the experiment be successful, two others will take their places. It is hoped that in this way the elements of sanitary science may be impressed upon the Maories, and that the nurses may be able to prevent, in a small degree, the rapid spread of phthisis and other diseases decimating the native population so sadly."

"Do any of the hospitals take paying patients?"

"Yes, a few; but it is rather a vexed question."

And then, expressing the hope that Mrs. Neill would much enjoy her six months' holiday, I rose to go.

"I shall be very glad to do so," she rejoined, "as after thirteen years' absence from England I feel I have earned it. But I have been here a month, and have worked hard nearly all the time. However, I daresay that will mend shortly, and I am not one of the people who like to rust out."

### Appointments.

Canterbury Sanatorium.—On April 1st Miss Charlotte Bray was appointed Matron. She was trained at the Royal Infirmary, Newcastle-on-Tyne, for two years, at the Hull Sanatorium for one year, and at the Liverpool Hospital for Diseases of Women for one and a half years. Since then she has been for two years senior sister at the Buckingham Infirmary, Aylesbury; sister for two years at the Cancer Hospital, Brompton, London, S.W.; and matron of the Emsworth and District Cottage Hospital, which post she has retained for the past three years.

District Cottage Hospital, Ulverston.—Miss Agnes Tyson, the new Matron, asks us to say that she worked in the Queen's Home, Hull, for over two years, and that her training for district work was in the Battersea Home, London.

### The Royal National Pension Fund for Nurses.

### PRESENTATION TO MISS BURNS.

As our readers are already aware, the announcement of the forthcoming marriage of Miss Burns, daughter of the late Mr. W. H. Burns, chairman of the Pension Fund, with Mr. L. V. Harcourt called forth a spontaneous desire on the part of the members of the Pension Fund to offer their congratulations to her and to express their good-will towards her and her family.

We received a request from the nurses that a subscription list might be opened in the columns of THE HOSPITAL, and that we should undertake to carry out their wishes. To this we gladly assented, stipulating that the congratulation should be conveyed in the form of an address and that the contribution of each nurse should be fixed at sixpence. In making these provisions we were actuated by a regard for what we felt sure would be the wish of Miss Burns herself. We arranged further that the list should remain open for a limited period only, as it was only intended that those who signed the address should act as representatives of the whole body of policy-holders in the Fund. When we received 300 names we closed the list, only to reopen it to admit the names of one or two policy-holders from the colonies and abroad, who would have been sadly disappointed not to be included. The letters we received from the contributors contained the heartfelt expressions of recognition of the splendid and disinterested services rendered to the Pension Fund and to the whole nursing profession by Miss Burns's family. The main tenor of these expressions have been embodied in the text of the address, which runs :—

THE ADDRESS.

"The undersigned nurses of the Royal National Pension Fund for Nurses desire to offer to Miss Burns their hearty congratulations and sincere good wishes on the occasion of her marriage with Mr. Lewis Vernon Harcourt. The present position of the Royal National Pension Fund and its magnificent success testify to the wisdom and devoted care which have been bestowed upon it; and the nurses of the Pension Fund take the opportunity the present auspicious occasion offers to assure Miss Burns that they can never forget their indebtedness to her grandfather and to the members of his family, nor the splendid services which her father, the late Mr. Walter Hayes Burns, rendered to the Fund as chairman of its council from the day of its foundation."

We entrusted the framing of the address to Miss Beatrice Cameron, of George Street, Hanover Square, who is well known for her artistic and original work. She took the utmost pains to do justice to the occasion, with the result that a most tasteful and appropriate encasement was produced. The illustration will show the design, which was suggested by an old Italian well-head. It has been carried out in a beautiful green polished wood, relieved by gold ornamentation. The doors which enclose the address form frames for photographs of Watts's celebrated pictures, "Love in Life" and "Love in Death," printed in a beautiful rich brown tone. The address itself is nicely illuminated, and by the side of it hangs on a tiny hook a little brown book, in which are inscribed the names of the contributors representing the Pension Fund. Apart from the special interest which attaches to it, the whole forms a charming and decorative work of art. Nurse Walker, the first nurse to receive her pension from the Fund, appropriately signed the address for the contributors. When all was completed, Miss Burns kindly arranged to receive a small deputation of nurses to present the congratulatory address on Friday, May 12th, at her residence, 69, Brook Street, Nurses Walker, Ayrton, Fisher, and Connell, representing some of the earliest members of the Fund, forming the deputation. On assembling at Brook Street they were received by Mrs. Burns, Mr. Walter Burns, Miss Burns, and Mr. L. V. Harcourt. The Hon. Secretary of the Junius S. Morgan Benevolent Fund introduced the nurses, and briefly explained the nature of the offering and the reasons which prompted it. Nurse Walker then handed the address to Miss Burns, and Nurse Ayrton, the second nurse to join the Pension Fund, presented to her the little book containing the names of the contributors. A few moments were spent in examining and admiring the pretty offering, and then Mr. Harcourt, in the most delightful manner, addressed the nurses. He said:

MR. HARCOURT'S ADDRESS.

Miss Burns desires me to endeavour to express to you the great gratitude she feels for the beautiful presentation you have made to her to-day. I do so with great diffidence, for the relationship which alone justifies me in doing so is, alas! at present prospective and not actual, but that fact will not,

I hope, detract from my interpretation of her sentiments. She feels deeply your kindness to her to-day—a kindness which seems only to be warmed and softened by your constant contact with sorrow. She bids me say that she realises and appreciates that this presentation, though made directly to her, is prompted still more by your loving memory of her father and grandfather and by your affection for her mother, who have done so much by their generous and thoughtful aid to relieve those who have devoted their lives to the service of suffering humanity from the most carking of all cares, the anxiety for their future. Both Miss Burns and I have had, in our lives, the misfortune to require and the good fortune to secure the services of trained nurses. We have known what the presence of these ministering angels mean in a house; we have ourselves seen how a nurse will come into a sick room a stranger, and has not time to leave it before she has become a friend. Their presence is the sunlight of humanity, and their voices are the music of the world. You, ladies, have robbed sickness of its suffering, and pain of half its pangs. You have learnt that wonderful lesson which is taught to so few, of how to quicken convalescence and how to soften death. You are the silent contributors to the triumphs of science; medicine depends on your watchfulness for its diagnosis, surgery on your fidelity for its success. This touching address, and the beautiful casket in which it is enshrined will be a chief ornament and treasure in the new home we are about to establish, and we shall hand it down to those who come after us as a token of the esteem which Miss Burns is so proud to have deserved, and still more as an encouragement to them to follow in the foot-steps of their predecessors in lending all the aid in their power to the maintenance and encouragement of those who have enlisted their lives and hopes in one of the noblest professions the world has ever seen.

The close attention paid during the delivery of Mr. Harcourt's words, and the murmurs of approval, showed how they went to the hearts of the hearers. Mr. Walter H. Burns also addressed the nurses on behalf of himself and his mother, and he in a most feeling manner alluded to the gratification which it gave him to find the memory of his father still cherished by those for whom he had so devotedly worked. He said he was proud to feel that his father and Sir Henry Burdett had done more than anyone else towards establishing the Pension Fund. Mr. Burns' words were all that was needed to assure the nurses of the Pension Fund that the noble and disinterested spirit which had secured for them the munificent assistance and invaluable and devoted personal service of two generations of the same family actuated those who remained to uphold the honour of honoured names of Burns and Morgan. Mr. Burns' words closed a simple but impressive little ceremony fraught with a significance beyond the outward expression of the feelings it represented. Only twelve years ago no pension fund for nurses existed, nor would one probably have existed, had not Mr. Junius Morgan come to the aid of Sir Henry Burdett and helped to found it. Neither would the magnificent Fund which represents the savings of 4,000 nurses have been in the sound financial position of to-day had Mr. Walter H. Burns, his son-in-law, not given continuous and devoted attention to the conduct of every detail in the organisation, notwithstanding the many calls upon his valuable time. But the deeds of such men do not die. They live to inspire similar actions, and to warm and soften the hearts of thousands with feelings of loving thankfulness and gratitude. This little ceremony at Brook Street was the beginning of a harvest reaped from the seed sown but a few years ago. After the presentation the nurses took tea at Mrs. Burns' invitation, and thus concluded the interesting event. The little party was destitute of all formality, and when it broke up all felt they had spent a most pleasant afternoon. The kindness of the reception they met with will not easily be forgotten by the nurses.

### "Don't."

### A FEW HINTS TO NEW "PROS."

Don't have one single opinion of your own.

Don't take a doctor round the ward by yourself.

Don't—to a senior—speak till you are spoken to.

Don't forget to open the door for any of the staff.

Don't forsake your patient in the anæsthetic room.

Don't ask "What is the matter with that patient?"

Don't mistake the assistant matron for a fellow "pro."

Don't forget to have your cap starched before wearing it.

Don't when requested to bring a spatula take the poultice-knife.

Don't ever forget that you "know nothing" about anything.

Don't ever go near a doctor or a student unless absolutely obliged.

Don't ever kneel down when on duty, not even to save your life.

Don't take offence if your friend the porter gives you fatherly advice.

Don't ask questions in a ward during the early morning sweeping and dusting.

Don't arrive at the hospital with three or four dress baskets, a guitar, and a tennis racquet.

Don't, when in doubt in a surgical ward, forget to say either "blue wool and gauze" or "spread boracic."

Don't break your heart if your milk boils over in the ward kitchen, and the wardmaid declares "She'll never blacklead that there stove again!"

Don't be surprised if, after having polished brasses till you have nearly skinned your fingers, someone says to you, "There are those brasses still to clean!"

Don't forget that it is—

"Yours not to make reply,
Yours not to reason why,
Yours but to do and die."

Don't, when a new nurse appears upon the scene, and you are no longer a junior, forget that once you, too, were only a "new pro.," and do to her as you would had been done to you.

### Echoes from the Outside World.

AN OPEN LETTER TO A HOSPITAL NURSE.

I WAS lucky enough to get a peep of the Queen on Monday. I had to do some shopping, so I started a little earlier than I should otherwise have done, and got down to the Albert Memorial very comfortably, all things considered. Of course, there were heaps of people, but they were all most quiet and well behaved. It was quite easy to get standing room, and we all had a fairly good view. There is no use saying the Queen looked very well, because you must have read it a dozen times already. Everyone who has seen her during her visit to London this time—and everyone who has not—says the same thing, but still it is a fact. Only she looked very black as to garments, for even the rosette of white tulle, or the little white feather she wears on important occasions, was absent. I fancied I saw her turn and look at the statue of her husband as she passed. I have always fancied that the Albert Memorial is one of her favourite monuments.

DON'T forget all the events for the 24th. The two thanksgiving services at St. James's Chapel and St. Paul's Cathedral, and the opening of most of the rooms at the Queen's old home at Kensington Palace to the public. I should not be surprised, also, if the illuminations in the evening—of which there are always a certain number on Her Majesty's birthday—are not much more general this year.

SURELY there is a magic in the name of Paderewski! I determined to go and hear the great master on Tuesday, and I concluded that if I got to St. James's Hall a couple of hours before the performance began I should stand the chance of securing a capital seat. Yet, when I arrived, I found the staircase blocked up with people, picnics in full swing, studious ladies reading wise treatises or small text books, frivolous females deep in yellow-backed novels, and a most annoying air of "been here for ages" written large on the countenances of those who were nearest the door. But I got into the Hall all the same, and had a lovely time. The place was densely crammed, and here and there I noticed a nurse's bonnet; but men were extremely rare. The programme was all Beethoven and Chopin. Amongst the former was the "Appassionata" Sonata, which seemed the perfection of passion, peace, and restlessness, all following each other in quick succession, so that even the disturbance caused by a lady who fainted badly and had to be carried out could not quite dissipate the feeling of happiness it gave one to listen to such music. But I did wish the poor lady could have waited till the interval. Paderewski, who looked exceedingly well, was in a most generous mood, and though the programme was exhausting, he gave us one of Liszt's rhapsodies and a waltz of Chopin's as extras. We were all very grateful, and went away more in love than ever, if it were possible, with the Polish pianist.

POOR "savages" at the South African show! Whether or not it was wise to bring the Zulus, Swazis, Kaffirs, and Matabeles over here at all is not a matter I need discuss. But having made up their minds to leave their own land and their own people, and having traversed so many thousands of miles, I can well understand that the poor simple savages revolt at what they feel is fearful unkindness on the part of the authorities, namely, the refusal to grant them permission to go outside the walls of the Earl's Court Exhibition. Even the big wheel they have only gazed at from below, though most of them have expressed a strong desire to mount as high as they could and thus get at least a glimpse of the land which according to their ideas was promised them, indeed, but which they may not enter. The crowning point of their misery appears to have been reached when they realised that the great White Queen, as they call her, who is to them a very wonderful person, was coming into the capital quite close to them, and yet they might not look upon her face. But it is very difficult for the officials to know what to do. They are only too well aware that if once these natives are allowed to wander forth at their own sweet will the very first thing they will do is to find their way to a drinking bar. After that their doom is sealed. A couple of glasses of beer would hopelessly intoxicate them, and, unfortunately, they have the wherewithal to purchase drink or any other harmful luxury. The public, by way of showing their appreciation of the "fine fellows," give them frequent tips, which are seized on with much pleasure by the recipients, but lead to a good deal of trouble, because the possession of coin is naturally followed by a desire to spend it. Arrangements have been made to hold Sunday services in the African village. Perhaps the management hope by this means to soothe the savage breast, though I do not know whether music forms any special part of the devotion of the natives.

I KNOW girls who have been very enthusiastic on the subject of nursing in their own homes become very downhearted and inclined to grumble—in private—when they realised how much rough work falls to the share of a probationer. For the comfort of those who may be smarting a little just now, outwardly and inwardly, at the sore knuckles and reddened hands which are the result of industrious cleaning, I may remind them that nursing is not the only profession which has to be begun at the bottom. At the present moment there is a young lady at one of our London theatres who is a relative of a well-known peer, and who has had a fair amount of experience for a beginner, as she has been touring in the provinces. Yet she is perfectly content to play nothing higher than "walking lady" with the chance of better things some day. How soon that chance may come she does not know, as she is fortunate enough to be understudy to the leading lady. Still, for the *nonce*, she is only a stage "probationer."

CAN you tat? I rather doubt it, because it is not at all a modern accomplishment. Our grandmothers and great-aunts were splendid "tatters"—if such a word is allowable—but the work is too fiddling, too quiet, and too "genteel" to have seriously appealed to the present generation of go-ahead, scramble-through-life folks. But the fact that tatting is to be one of the most up-to-date trimmings of the year may make it worth while for some of us to get a lesson from an old-world friend, and spend our spare moments—if we have any—in making narrow trimming, lappels, and sailor collars of this once much-esteemed work. The advantage of tatting is that the implements required are so limited. A nurse can put her cotton and her shuttle into her pocket or her bag, produce it at any moment with no fuss, and go on working when even the light is dim. Five minutes now and then soon produces wonders, and in a few weeks a pretty little gift for a fashionable friend will daintily recline on a piece of pink paper ready to send as an offering "from a busy nurse." A still further point in favour of tatting for uurses is that it makes no noise.

HAVE you ever heard of "Parochial Marmalade?" It is neither the name of a new firm of manufacturers nor is it made from a new combination of ingredients. It is simply the name given to an excellent specimen of the article said to be "a substitute for butter," made by a lady at King's Langley, and sold by her for various Church charities. That there is money in marmalade cannot be denied, for the lady has cleared over £82 profit, and has made 11,000 lbs. of the breakfast delicacy in seven seasons. Two country cottages are utilised for the manufacture, and a good many oil stoves, together, of course, with a vast amount of energy and trouble. A very small stock-in-trade to produce such good results.

### Workhouse Nursing.

The following is the substance of a paper read by Miss O'Reilly, assistant secretary, Meath Workhouse Attendants' Association, at the recent Poor Law Conference, Malvern:—

The number of sick, infirm, and dying people to be found in our workhouses is greater than is generally known. In many cases the infirm are ineligible for the infirmary, and sometimes the sick and the dying are too ill to be removed there. Frequently, the only attendants are the wardsmen and wardswomen chosen from amongst the inmates, and it is not to be wondered at if they are found untrustworthy, ignorant, and otherwise unsuitable. Indeed, pauper nursing has nothing to excuse it, not even the plea of economy. Pauper attendants waste what they do not care for, and use for themselves the nourishment and stimulants ordered for the sick.

It is only occasionally that the abuses which arise from pauper attendants come before the public, but the amount of suffering it entails is still more seldom heard of or recorded. Quite recently the hon. secretary of a workhouse visiting committee wrote to us saying: "The present wardswomen and wardsmen are often very unsatisfactory, and the ladies' visiting committee of the —— Union, of which I am hon. secretary, would be very glad if they knew of any satisfactory way out of the difficulty." In one of the most important unions in the North of Ireland the staff provided for the nursing of 60 beds consisted of one so-called nurse (who had not regular hospital experience and teaching), and to assist her five pauper women, one of whom was about 65 years of age, five female pauper helps, and two male helps, both infirm. Besides these five women a wardsman was kept on the male side of the house; his age was about 74, and his hearing was defective. Previous to his coming into the workhouse he had been an ordinary day labourer. He was assisted from time to time by one or two old and infirm men who sat up at night with bad cases. One such was looking after a case of cancer on the day of the inspector's visit. The inspector also saw a third old man who occasionally acted as night nurse, and his qualification would appear to have been that he was feeble and had a paralysed arm. Many of these pauper attendants are subjects for nursing themselves.

Trained attendants in unions are needed, not only as nurses to the sick, but for purposes of supervision.

As a result of a recent midnight visit paid by two guardians to the Westminster Union Workhouse, a thorough inquiry is to be instituted into the conduct of nurses and others. The unexpected visitors found the gates separating the male from the female quarters unlocked, and one of the members declared he had discovered that it was a nightly occurrence for nurses and female inmates to go over to the males' quarters.

Here is another illustration of nursing in a Poor Law union. It is taken from an Irish workhouse, but I have learnt on good authority that similar cases occur at the present time in England. There were five cases of typhus in the union referred to. Three of these patients were delirious, the nurse was untrained. She was assisted by paupers during the day, she was replaced by paupers during the night. The nurse caught the fever and was buried within seven days. The dying nurse, who fell ill at her post, and the fever patients who had been under her charge, were "nursed" only by paupers. A trained nurse was obtained on the day of the funeral. There were now in the union five cases of typhus fever, two cases of scarlatina, and one case of diphtheria. The new nurse, after her day's work of anxiety and danger, felt obliged to go round to her patients' beds every two or three hours during the night. She frequently found the pauper attendarts asleep, but did not think they should be blamed, as the female pauper was supposed to cook and wash for all the patients and do the scrubbing work during the day; in fact, she was the only wardsmaid, and was supposed to do night duty as well. Under such a strain the nurse sickened of typhus and was found lying like a log on a bed of straw.

To ameliorate the condition of such workhouse inmates the Countess of Meath has founded an association to provide attendants for them.

At her own expense she has placed more than eighty young women as probationers in various hospitals and homes throughout the country in order to qualify them for the position of attendants in workhouses. Amongst the institutions in which they are trained are: The Crumpsall Infirmary, Manchester; Birmingham Children's Hospital; Kent and Canterbury Hospital; the General Hospital, Nottingham; the Royal Bucks Hospital, Aylesbury; the General Infirmary, Worcester; the Cheltenham Hospital; Moseley Convalescent Hospital, Birmingham; Poplar Hospital; Mildmay Memorial Hospital; St. Lucy's Home, Gloucester; St. Peter's Home, Kilburn; Home for Invalids, Highbury; St. Peter's Home, Woking; Meath Home of Comfort, Godalming.

The term of training usually lasts for one year. In some cases the year is sufficient, but in others it is considered advisable to give the probationer two years' training before she goes as attendant or assistant nurse in a workhouse.

Nearly fifty of these probationers have now obtained Poor Law appointments, and are in receipt of good salaries from the guardians, varying from £20 to £30 per annum. We do not find that these salaries correspond with the qualifications of the attendants appointed, but depend on the views taken by the guardians; and as the guardians are not always able to distinguish between certificates and testimonials, and in some cases between a trained nurse and one untrained, it is not surprising that we occasionally find that the attendants with shorter training are offered the highest salaries. This is a matter which we hope will right itself in time as the education of the guardians progresses.

Amongst the workhouses to which these attendants have been appointed are Islington, Whitechapel, St. Pancras, Poplar, Bow, Fulham, Shoreditch, Isleworth, Minster, Holborn, Blandford Eastry, Saffron Walden, Ouleston, Oxford, Ely, Chippenham, Hastings, Hungerford, North Walsham, Eastbourne, Pembrey, Leicester, Bristol, and Liverpool. Lately, however, these attendants have been placed in positions of such responsibility that the committee are considering the advisability of extending the training in all cases to two years.

From the many letters received from assistant nurses here are two which will give some idea of the work they are doing:—

"It is with much pleasure that I write to tell you a little about my work, as you so kindly wish to hear. On the whole I think I am getting on very well, and like my work very much. The first six weeks I was on night duty with the females, but now am on day duty with the males. We have at present thirty-six females and thirty-three females. Nearly all of them are very patient and grateful for anything done for them. Most of them are very old people, and some have to be washed and fed like little children. At present I have had nothing to do but what had been either practically taught or explained at St. Peter's."

"I am very sorry not to have written to you before, but we were so very busy I have had little time for myself. For the first seven weeks I was on night duty in the female infirmary, and owing to so many changes of nurses I have been on day duty in the male infirmary since September 29th. My duties are to go down to the workhouse morning and night to give medicines to any of the men that require it; then at eight a.m. I attend to all the out-patients, such as boys from the school and men from the house, who need poultices or wounds dressed. It is nearly nine a.m. before I can begin my work in the infirmary. First I have to wash all the helpless patients, make their beds, &c., take all temperatures, change all poultices, fomentations, and dressings. It is then time to change all crib cases before dinner. I then see all the dinners served and weighed for each patient, and then feed those that are unable to feed themselves, and after dinner I have to go all round again to each ward, nearly the same as in the morning; and after tea, to wash the patients and prepare everything for when the night nurse comes on duty at eight p.m. The work is very heavy just now. I did not mention that I have to attend on the casuals in the tramp ward. The doctor writes on the slate when any of them need medicine, poultices, or dressings done for them. I see more here in the way of nursing than I did at St. Peter's I have seen the doctor set three broken legs within the last few weeks; two of the men are getting on nicely, and the other poor old man only lived a week after; and what a lot of men we have brought in insensible through drink at all hours of the night. We have now eighty-six patients, and only myself and the charge nurse to do everything for them. One thing, I have no ward work to do; the wardsmen do that. I am pleased to tell you that I am enjoying very good health, which I think helps me to do all I can for the sick. I never mind how much work I have to do so long as I know it is to make them happy and comfortable, and to help them to bear their pain which God has seen fit to send them, and I know I shall have my reward by and bye. I can say that I am very happy and comfortable here; still I am looking forward to the time when I shall spend a few days at St. Peter's Home with the sisters. I often have nice letters from them, both interesting and encouraging, and seem a great help and comfort to me."

It is with much regret that the association learns from this nurse as well as from others that they have the casuals to attend, as there is danger of carrying infection from tramps to the children and sick, and even if the tramps themselves are not ill, it is most likely their clothes are filthy, and may probably bear the germs of many diseases from places through which they have passed. A certain amount of friction has sometimes arisen from the fact that our attendants have been called upon to work under an untrained matron, who, although she may be a worthy woman, is unsuited to be placed over a trained nurse. The following letters will give an example of this difficulty:—

"I have considered matters fully, and have quite made up my mind to leave here. If the matron was a trained person, I should certainly battle on and try and make all necessary improvements for the welfare of those put under my care, but as it is now, I should not care to do so. This is not a very large place, and the nursing is sometimes very light, but throughout the whole house there is no proper management or method. I shall certainly tell the 'Board' my reasons for resignation. My predecessor was also a trained nurse, but also failed to get a reform. She got thoroughly downheartened, and also made it unpleasant for herself."

"I do hope you will not think me ungrateful if I released myself for a time from the association, as under my present frame of mind towards workhouses I do not feel I could do justice to take another appointment when I leave here. I shall repay the fees, but in loyalty to you for your great kindness on my behalf, I shall give the association the year in a workhouse, but I must first have a change until I completely recover the dislike I have taken to workhouses. The doctor told me a few days ago that the matron called me her 'servant.' I said that was really what she required, not a hospital-trained nurse. The doctor told her as long as she treated nurses in such a manner no one would remain here, but she has been against a trained nurse coming in, so there rests the sequel—the manner in which we are treated."

"I have now passed my first week at the above and feel quite settled down; I like the place exceedingly well, everything is so entirely different, and the sick and aged are well provided for, which is my greatest pleasure. There are three nurses in all here, the head nurse has been here for 33 years, and is an exceedingly nice person, and makes us feel thoroughly at home. My fellow assistant, as you know, belongs to our association."

"You will be sorry to hear I resigned yesterday. I have had a most miserable time lately with the matron. She wanted to teach me my work—a woman who knows nothing about nursing. I am very sorry to have to leave here as I have got attached to my patients, and was in other ways very comfortable. The guardians asked me to withdraw my resignation, but I told them I could not with an untrained woman as matron. I do not know yet what I shall do, but if I do work under the Poor Law it will be where there is a trained matron."

The new Local Government Board order not only makes pauper nursing illegal, but prescribes that every nurse henceforth appointed must have had practical training. Nevertheless, the supply of nurses is so much less than the demand that untrained persons sometimes gets appointments. Dr. W. R. Davison, of Ballymena, mentions an incident in a letter in the *British Medical Journal* of April 15th of a woman who professed to be a nurse, and was recognised by the Local Government Board. I quote from his letter: "About the first thing I asked her to do was to put a carbolic compress on a bad leg, and this she did as follows: She boiled the pure acid, saturated lint in this, and bandaged it on the leg, with results which can be easily imagined. When told to wash a patient, she took him out of bed naked, and placed him on the floor in the ward; while as for bandaging, cleanliness, and tidiness, she knew nothing about them."

"At present there are about 40,000 sick and bed-ridden paupers, besides 20,000 aged and infirm, in the workhouses of England and Wales. These 60,000 are nursed by 3,715 women, of whom only 1,961 had previous training." (Quoted from the returns of 1896.)

The Countess of Meath, in founding this Association, aimed not merely at providing attendants for the aged, infirm, and young, but at introducing a higher moral and more cheerful tone in the sick wards, and the increasing demands from Poor Law Guardians for our attendants shows how much they are appreciated.

There have been many applications from men asking to be trained for workhouse attendants, and the association regrets that they cannot undertake this branch of work, for in many wards male attendants would be more suitable than women nurses.

In conclusion, Miss O'Reilly pointed out that the public could help in this work by sending to the association any young people who would be suitable for probationers, by giving information as to good training institutions in which to place them, and by bringing their influence to bear on local Boards of Guardians to appoint trained attendants where they are needed.

### The Medical, Surgical, and Hygienic Exhibitors' Association.

This exhibition will be held at the Queen's Hall, Langham Place, on May 23rd, 24th, 25th, and 26th, from 2 p.m. to 10.30. There will be music by a select orchestra every afternoon and evening. Nurses in full uniform will be admitted free. I hear there will be several interesting novelties, and, as usual, the Scientific Press will have a stall.

### Everybody's Opinion.

[Correspondence on all subjects is invited, but we cannot in any way be responsible for the opinions expressed by our correspondents. No communication can be entertained if the name and address of the correspondent is not given, as a guarantee of good faith but not necessarily for publication, or unless one side of the paper only is written on.]

### THE ARMY NURSING SERVICE RESERVE.

"A MEMBER OF THE SERVICE," writing from Scotland, says: I read your account of the Army Nursing Service Reserve in the last number, and would be obliged if any of your correspondents who may belong to it will tell me whether they find any difficulty in gaining hospital appointments on that account. I belong to the Reserve myself and so far have not done so, but I have been told by others that the fact of our being liable to be called up stands in the way of our getting good hospital posts.

### "DEARTH OF TRAINED NURSES."

"LILY" writes: I am pleased to see, and fully endorse, "Bluebell's" opinion that trained nurses are not paid enough. It does seem a shame, when we give up our strength and time, that we are not treated less like machines for making money, and are only given a paltry salary of £25 a year; more often less. Cooks and butlers frequently get £35 and £40 a year, and yet they have no care, no anxiety, no lives depending on their watchfulness as a nurse has. Except for the love of the work—which most nurses have—there is very little to induce anyone to remain a nurse. "Bluebell" is quite right when she says there is no thought for the poor, tired-out nurse on her return to the institution from a case where she may have had a great deal to try her. I myself have often had to go direct from one case to another and not had even a night's rest between. If some of the superintendents had a little more feeling, how very different our lives might be. They forget they have been nurses themselves, which should make them more thoughtful for those under their care.

"ANOTHER NURSE" writes: In reference to a letter signed "A Nurse," giving her experience of "Homes for Trained Nurses," I should like to add my testimony to the other side of the question. After my training I joined a nursing institution, which bore an excellent name and had a staff of over one hundred nurses. The salary was £30 clear, with a bonus of 10 per cent. after the first year, and a liberal supply of good uniform. The matron was a lady of high ideals, but with plenty of practical commonsense. She was the friend of all, and often I have found her kind advice and sympathy invaluable in times of difficulty. Of course, if one fell short of one's duty, then no one could be more severe than she (as I knew once to my cost). The home was made as home-like as possible, rules were few, and a tired nurse could even breakfast in bed, if waited on by a fellow-nurse; and for sickness a small cottage was kept ready for our use, and all expenses paid. Last year, whilst in town, I visited a friend working on "The Co-op." She was lodging in one room at the top of a high house, in absolute freedom certainly, but at what a loss in comfort! We compared notes, and agreed that though she earned more money, yet her expenses were so heavy that there was little difference in the long run. My main object in attempting this defence of nursing institutions is for the sake of any nurse who may contemplate joining a "home," lest she might be frightened away by "A Nurse's" experience. To such a one I would say, choose a good institution, and I do not think you will regret it.

### NURSES' HOLIDAYS.

"INASMUCH" writes: I have been a constant reader of your valuable paper for many years past. Will you permit me to say a few words about nurses' holidays and times off duty? I feel sure there must be very many like myself who have lost all their near relations and have no one with whom to make a home when they are not at work or during a holiday, and to whom the "has-to-be-paid-for-holiday" is not only an expense, but a real burden. District nurses especially do not receive large salaries, and find board and lodging costly, as they are not able to economise for themselves. I often think of the many well-to-do convalescent patients whom the doctor insists upon sending away for change of air, some of whom, perhaps, would only be too thankful for the companionship of a nurse when she did not require a fee. I do not mean that nurses are to be burdened with sick patients or patients just recovering from sickness during the time they call holiday; but there are cases when next door to no actual nursing is required, and the nurse could enjoy the rest and change, and perhaps come back looking better than she has done for years. Then, too, there are ladies living in the country who might receive a nurse for change and rest without causing any discomfort to her household. I am not asking for charity, but I cannot help thinking that there are many ways like this, in which we all might help each other.

### The Sussex County Hospital.

### OPENING OF THE NURSES' HOME.

ON Saturday the new home for the nursing staff at the Sussex County Hospital, Brighton, and the covered approach connecting it with the hospital were formally opened. An account of the home has already appeared in these columns. The home is connected with the main building by a covered way, leading from the corridor on the first floor. This is the gift of a lady, the wife of a very generous friend to the Sussex County Hospital.

In lovely weather, about three o'clock, many friends of the institution arrived, and were conducted by the stewards to the terrace in front of the new home, those officially connected with the ceremony being received in the board room by the chairman, Mr. Herbert Fisher, and members of the board of management. On the arrival of Lady Louise Loder, who had kindly consented to open the building, a procession was formed, the vicar of Brighton (the Rev. Prebendary Hannah) and the Rev. L. H. Orton (chaplain of the hospital) leading the way up the covered approach, followed by the chairman and Lady Louise Loder, Sir Henry Harben, Mrs. Fisher, and many others. On arriving under the verandah outside the home Lady Louise was presented with a lovely bouquet of pink roses by the matron on behalf of the nursing staff. Psalm cxxi. was then sung, the nurses leading the singing, and Miss Scott, the matron, presiding at the piano. A few appropriate prayers were said by the Vicar of Brighton, the religious ceremony being brought to a close by the singing of the hymn, "Now Thank we all our God." Lady Louise having proceeded to the entrance, unlocked the door with a silver key presented to her by the chairman on behalf of the committee of management, and then, in a few well-chosen words, declared the building open. Sir Henry Harben, under whose presidency the building was begun last year, made an excellent speech congratulating the company on the completion of a scheme which had long been desired by those connected with the hospital, the accommodation for housing the nurses having been utterly inadequate, "some being housed at the top of the main building, some in the basement, while others slept in two houses outside the walls." He next spoke of the privilege of giving, and urged them to do all in their power to help in paying off the debt, and to further in every way the completion of the improvements which still remain to be carried out. The Chairman thanked all those present for their attendance and kindly interest in the work. The home being thrown open, many visitors availed themselves of the opportunity of inspecting the building, and expressions of delight at the completeness of the arrangements were general. An excellent tea was provided in the nurses' dining hall, where all were cordially welcomed by the matron and well looked after by the staff.

### Minor Appointments.

Bath Union Infirmary.—Miss R. B. Fry has been appointed Superintendent of Nurses at the Bath Union Infirmary. Miss Fry was trained at the Devon and Exeter Hospital, and was on the hospital staff and the private nursing staff till September, 1897, when she was appointed out-patient sister. She is the fourth sister from the hospital who has been selected during the year as matron for a hospital or convalescent home.

Rossal School, Fleetwood.—Miss Jeanie Morrow has been appointed Nurse-Matron of the infectious hospital of this institution. Miss Morrow was trained for three years at the Belfast Royal Hospital. Subsequently she held an appointment as district nurse of the Manchester and Salford Sick Poor and Private Nursing Association, affiliated with Q.V.J.N.I.

The Passmore Edwards Cottage Hospital, Liskeard.—May 2nd: Miss E. Linton, Nurse Matron. She was trained in the General Infirmary, Burton-on-Trent. Her previous appointments were private nurse for two years, Nottingham and Notts Association; charge nurse at the Birmingham and Midland Ear and Throat Hospital, and district nurse, Gateshead.

Canterbury Workhouse Infirmary.—May 16th: Miss Ellen Connelly, Superintendent Nurse. She was trained at St. Olave's Union Infirmary. Her previous appointments have been probationer and staff nurse, St. Olave's Union; charge nurse, Cardiff Union; charge nurse, City of London Union; and private nurse abroad.

Union Infirmary, Watford.—Miss Annie Mercer has been appointed Assistant Nurse at the Union Infirmary, Watford. She was trained by the Meath Workhouse Attendants' Association.

### Presentations.

A pleasing ceremony took place at the Gravesend Hospital last week. It has been the practice of the hospital to give a bronze medal to their nurses for three years' service, a silver medal for five years' service, and the committee lately resolved to give a gold medal to all nurses who complete ten years' service. Nurse Kathleen Quiggin is the first nurse in the institution who has served the full term of ten years, and the chairman, Mr. W. Tingey, expressed his desire to the committee of the institution to present the medal at his own cost. Accordingly, in the presence of the nursing staff and in a few well-chosen remarks, expressing gratification at the good work the nurses are performing in the institution, and particularly referring to the punctiliousness, kindness, and devotion of Nurse Quiggin to her duties, Mr. Tingey made the presentation. Accompanying the medal was an artistic illuminated certificate on vellum, of which the following is a copy: "This is to certify that Kathleen Quiggin in September last completed ten years' service as nurse in this institution, and in recognition of her faithful services during that period I have, with the permission of the committee, presented her with a gold medal.—William Tingey, Chairman." The certificate contains the arms of the Borough of Gravesend in the centre, with a view of the hospital in each corner, namely, the institution in its primitive state when first opened, the children's ward, the Russell wards, and the hospital in its present improved condition. The hon. secretary, on behalf of Nurse Quiggin, thanked the chairman for his kindness, and said that, although it was a testimonial bestowed upon Nurse Quiggin in particular, all the nurses felt it an honour conferred upon the whole staff.

### For Reading to the Sick.

### WHITSUNTIDE.

"And they were all filled with the Holy Ghost and began to speak with other tongues as the Spirit gave them utterance."—*Acts* ii. 4.

Our blest Redeemer, ere He breathed
His tender, last farewell,
A Guide, a Comforter, bequeathed
With us to dwell.

He came sweet influence to impart,
A gracious, willing guest,
While He can find one humble heart
Wherein to rest.

—*Hymns Ancient and Modern.*

**Reading.**

Jesus Christ's manifestation of God was completed in His revelation of the Spirit. He promised that He would send forth the Spirit from the Father upon men. "It is expedient for you that I go away, for if I go not away the Comforter will not come unto you." As a fact of history, and as a matter of experience, the promise of Jesus Christ has been fulfilled. He left the world in visible presence in the descent of the Holy Spirit on the Day of Pentecost. "When the Day of Pentecost was now come they were all together in one place, and suddenly there came from Heaven a sound as of the rushing of a mighty wind, and it filled all the house where they were sitting, and there appeared unto them tongues, parting asunder, like as of fire, and it sat upon each of them. And they were all filled with the Holy Spirit."

That the advent of the Spirit was an actual fact is attested by the marvellous change which passed over the characters of the Apostles. Previously timid and faint-hearted, they suddenly exhibited signs of supernatural courage, bearing their witness to Jesus Christ far and wide in the face of ridicule, persecution, and death. "The boldness of Peter and John" was a matter for marvel to the Jews. The Apostles "spoke the Word of God with boldness."

Twelve unlearned and ignorant men went forth to found the Christian Church, and to convert the world. History testifies to their success. The Christian thinker attributes the marvel to the coming of the Holy Spirit, which Christ promised. "Ye shall receive power when the Holy Ghost is upon you; and ye shall be My witnesses both in Jerusalem and in all Judæa and Samaria, and unto the uttermost parts of the earth."—*Vernon Staley.*

Holy Spirit, Heavenly Dove,
Now descending from above,
Breath of life and fire of love,
Hear us, Holy Spirit.

Source of strength, of knowledge clear,
Wisdom, godliness sincere,
Understanding, counsel, fear;
Hear us, Holy Spirit.

Comforter, to whom we owe
All that we rejoice to know
Of our Saviour's work below,
Hear us, Holy Spirit.

Come to strengthen souls who yearn
More of truth divine to learn,
And with deeper love to burn;
Hear us, Holy Spirit.

Keep us in the narrow way,
Warn us when we go astray,
Plead within us when we pray;
Hear us, Holy Spirit.

Holy, loving, as Thou art,
All Thy sevenfold gifts impart,
Never more from us depart;
Hear us, Holy Spirit.

—*Hymns Ancient and Modern*

### Travel Notes.

By Our Travelling Correspondent.

### XXIII.—CARLSBAD AND FRANZENSBAD.

These celebrated health resorts are so near together that I think we may consider them in one article, the same information as to the expenses of the journey and hotels applying nearly equally well to either.

### The Journey.

The return tickets only last for thirty days, not long enough for the cure, but arrangements may be made to extend the time. The cheapest way is by Harwich and the Hook of Holland *via* Cologne, Frankfort, and Nuremberg. This costs £5 10s. 8d. first-class and £3 17s. 11d. second. By the Harwich and Antwerp route it is about 3s. dearer. The expensive way is as usual by the short sea voyage *via* Dover and Calais, £6 14s. 2d. first-class and £4 17s. 11d. second. If fatigue is to be avoided you might sleep the first night at Cologne, the second at Wurzburg, and the third at Nuremberg, all interesting places, and here the trains fit most conveniently. At Cologne go to the Hotel Continental, close to the south portal of the cathedral. It has a lift and is reasonable. At Wurzburg, to the Russischer Hof, because it is near the station. At Nuremberg you will probably remain some days unless familiar with its charms. Go to the Bayrischer Hof or to the Goldner Adler.

### Carlsbad Itself.

Here you may undergo complete hydropathic treatment, and the waters are recommended for a large number of complaints—mostly those connected more or less with the liver and stomach. Almost all forms of dyspepsia are benefited by the treatment, and it has long been considered the place of all others for gout. You would not, of course, go without careful medical advice. The usual plan is for your doctor in England to forward you, if one may use the expression, to one of the Carlsbad doctors, and he indicates to you what treatment to pursue. The season lasts from May till November, and during that time no place is livelier. The hotels are good, though somewhat dear, like all other fashionable places possessing the curative properties. It is well to write beforehand to secure rooms, for so great is the press in the height of the season that you are likely to come off badly if you leave this important matter till your arrival. Apartments are not difficult to obtain, and if extreme quiet is desirable they are preferable to the gay life in an hotel.

### Amusements and Advantages.

First and foremost there is a very good reading-room, two florins monthly. There are plenty of papers and a room for ladies, which is a great convenience. There is a very fair theatre throughout the season, and a capital band plays every day from six to eight at the Sprudel Spring, and at the Mühlbrunnen; the staff of musicians is sufficiently large to divide and thus be in both places. Sprudelstein wares, which a friend of mine designated as "loathsome souvenirs" are to be bought in great abundance, if there is anyone so benighted as to like such horrors. There is a good English church service twice a day, Presbyterian service in the Curhaus, and of course the Roman Catholic church. I am told there are great educational advantages, good school and first-class governesses and tutors, often an enormous comfort when there are young people, to whom the lazy gossiping life of a health resort of this kind is anything but beneficial.

### The Springs.

Their name is legion, and my space will not permit me to say much about them. The most important are the Sprudel, the Felsenquelle, the Mühlbrunnen, and the Marktbrunnen. Almost all are provided with colonnades, in which those who are taking the waters may walk in wet weather, a good deal of exercise being an important factor in the cure. The routine begins as early as five a.m., the crowd is prodigious, and waiting is somewhat wearisome before one can be served with one's dose of water. In the Alte Wiese are the best shops and plenty of "loathsome souvenirs." A favourite walk for those who are somewhat feeble is along the Alte Wiese, the Goethe Platz, and the Pupp'sche Anlagen, because it is on the level.

### Excursions.

The environs of Carlsbad are very beautiful, woods and mountainous hills abound, and if able to walk you will

Summit of the Herschen Spring.

thoroughly enjoy the scenery. For those who cannot do so, carriages are to be had at fairly reasonable rates, and donkeys for such expeditions as to the Hirschensprung and the Dreikreusberg. An agreeable excursion is to Dallwitz on the Eger, distant about three miles; here are beautiful oak woods and a porcelain factory. Another, a little further off, is to Hans Heiling's Restaurant, from which you see the romantic rocks on the Eger bank. Engelhaus, on the Prague road, is worth visiting for the sake of the scenery one sees on the road, though Engelhaus itself is not very striking.

### Franzensbad.

This is at a higher level than Carlsbad, being 1,900 feet above sea level. I think the scenery and surroundings are preferable to those at Carlsbad, and it is not so crowded,

The waters are like those of Carlsbad, good for gout and dyspepsia, but more especially suitable for female diseases, anæmia, neuralgia, &c. The great feature of the place is its mud baths. There are three bathing establishments, all excellent, and, as at Carlsbad, everything is done to make the time pass pleasantly. The Curhaus has reading and billiard rooms, smoking and gaming rooms, and a good orchestra. The inhabitants proudly record that they are visited by 10,000 patients yearly. I much prefer Franzensbad to Carlsbad, but personal preference has generally little to do with one's residence at either place, for usually one is with an invalid, or at any rate with some one supposed to "enjoy" bad health. There are, indeed, very sad sights at both places, but it is comforting to reflect that the 10,000 or 12,000 so-called invalids are by no means at death's door, and appreciate life thoroughly. There are many who consider a hydropathic course as indispensable as a spring clean in their houses, and accordingly one encounters a good many robust invalids. The woods and park round Franzensbad form charming promenades for those not equal to much exertion, and further a field there is the Castle of Seeberg distant some three miles, Antonienhöhe, a fairly high mountain; the Rammerbühl, and many others. It is worth while to visit Eger; there is a good deal of interest to be seen. Wallenstein was murdered there in the Stadhaus. The castle, too, stands grandly on the fortifications, though now only a ruin. The huge tower was built prior to 1183.

### TRAVEL NOTES AND QUERIES.

RULES IN REGARD TO CORRESPONDENCE FOR THIS SECTION.—All questioners must use a pseudonym for publication, but the communication must also bear the writer's own name and address as well, which will be regarded as confidential. All such communications to be addressed "Travel Editor, 'Nursing Mirror,' 28, Southampton Street, Strand." No charge will be made for inserting and answering questions in the inquiry column, and all will be answered in rotation as space permits. If an answer by letter is required, a stamped and addressed envelope must be enclosed, together with 2s. 6d., which fee will be devoted to the objects of the "Hospital Convalescent Fund." Any inquiries reaching the office after Monday cannot be answered in "The Mirror" of the current week.

BRITTANY (Berrie).—As a member of the C.T.C. you have nothing to pay to take your machine into France. But at the Customs you must supply yourself with a *permis de circulation*, cost 60 centimes. This is a new regulation to assist foreign cyclists in France. It is also compulsory that you have on your cycle a metal plate with your name and address. On the whole, I think you could not do better than make your headquarters at Quimper, in Finisterre. The only drawback is that it is a very long distance from your landing-place, and your time is short. I should train from St. Malo to Morlaix. It is a pity not to see Morlaix, one of the most typical towns in Brittany; stay one night there, and cycle to Carhaix, 30 miles. You are now in the best scenery in Brittany; sleep one night, cycle on to Quimperlé, taking Le Faouet en route, a run of 40 miles. You might stay there in preference to Quimper possibly. The coast scenery between Quimperlé and Quimper is the most noted in Brittany. The alternative is to go from Morlaix and thence to Quimper, but from Laudermeer it is too long for one day—quite 50 miles. Douarnenez, Audierne, and Pointe du Raz are all fine and all attainable from Quimper. English money cannot be changed in South Brittany.

VENICE (Khartoum).—Either spring or autumn there is delightful. Summer is hot and airless, and there is a good deal of smell from the side canals. The Hotel Danielli is a little beyond the terms you mention, but would probably come to your arrangement if you stay any length of time. It is the nicest of all the hotels, I think, for situation.

ROUTES TO ROME (Poppy).—You have a great choice of routes, and the difference in cost is but trifling. Dover-Calais, Laon, Milan, and Florence, first-class, £10 14s. 2d.; the same route branching off to Genoa and Pisa, £10 12s. 8d.; viâ Dover-Calais, Paris, St. Gothard, and Florence, £10 18s.; viâ Harwich, Brussels, Strassbourg, and Florence, £10 1s. 2d.; viâ Newhaven and Dieppe, Paris, Turin, Genoa, and Pisa, £9 0s. 4d. The most expensive route of all is by the Riviera, £11 10s. 3d.

LOWER BRITTANY (Stella).—Entirely unsuited to an invalid. Accommodation is far from luxurious, and sanitation leaves much to be desired. I think you would meet with all you want at Dinard. It is rather expensive, but proprietors are generally willing to meet the wishes of those who make a long stay. Try the Grand Hotel de Dinard. Yes, there is an English church open all the year. There is a very considerable English and American colony.

SIENA (Linguist).—Yes, you are quite right; it is said to be the purest Italian. Living is cheap at Siena in one of the native pensions, but you must not expect any great luxury or refinement. 2. At Perugia there is a first-rate hotel kept by an English woman, called Grand Hotel Perugia. Expensive for a short time, but reasonable for a week or fortnight. Choose your weather for visiting Assissi. If there is a mist you will see nothing of the pictures.

*(For Travel Advertisements see Page xv.)*

### Notes and Queries.

The contents of the Editor's Letter-box have now reached such unwieldy proportions that it has become necessary to establish a hard and fast rule regarding Answers to Correspondents. In future, all questions requiring replies will continue to be answered in this column without any fee. If an answer is required by letter, a fee of half-a-crown must be enclosed with the note containing the enquiry. We are always pleased to help our numerous correspondents to the fullest extent, and we can trust them to sympathise in the overwhelming amount of writing which makes the new rules a necessity.

Every communication must be accompanied by the writer's name and address, otherwise it will receive no attention.

*Convalescent Home.*

(71) Could you let me know if there is a convalescent home at Ilfracombe, Clovelly, Barnstaple, or Bridgewater?—*Nurse Elenor.*

We do not know of a home at either of these places. There is a small home at Morthoe—the Glenwood Convalescent Home—in connection with the North Devon Infirmary, for men, women, and children. There is the Royal West of England Sanatorium at Weston-super-Mare, and at the same place a convalescent branch of the Bristol Hospital for Sick Children and Women and the Bristol Medical Mission Convalescent Home. For other homes in the West of England see "Hospitals and Charities," Scientific Press.

*Breach of Contract.*

(72) As a matter of vital importance to the whole nursing profession I wish to lay the following case before you, and to solicit your advice. I was engaged by a lady for a month, commencing March 20th, but her case coming forward on February 7th she sent for me on that date. Being then engaged on another case I was unable to attend, and not having subsequent opportunity of engaging myself elsewhere for the month in question—although I could have done so on a previous occasion—my loss amounted to some four and a half guineas. I have both interviewed and written to the lady in question with reference to some compensation, but have not been able to obtain any redress, although I am informed she is well able to pay. I now wish to know whether I have a legal claim for the full amount, and if so whether the case if brought into court would undoubtedly go in my favour?—*Monthly Nurse.*

In such a case the nurse is, we believe, entitled to half fees. As to the result of a legal action it is impossible to give an opinion, and you should consult a solicitor.

*Truth's Doll Show.*

(73) Will you kindly inform me when and where the next *Truth* Doll Bazaar is to be held. May foreigners compete for and gain a prize, and of what nature would it be?—*E. M. L.*

*Truth's* Doll Show is usually held a few days before Christmas in the Albert Hall. The prizes are handsome and varied. The Editor of *Truth*, Carteret Street, Queen Anne's Gate, S.W., would be pleased to give full information to intending competitors.

*Convalescent Homes.*

(74) Could you please tell me if there are any nurses' holiday homes or a convalescent home where first-class patients are taken in at Llandudno? If so, kindly give addresses and oblige.—*Nurse.*

We are unable to recommend private nursing homes; but you should be able easily to obtain the information you need from the local medical men or from the newspaper.

*Rheumatism.*

(75) Would you kindly let me know if there are any places on the continent where my brother, who has rheumatism, would be likely to benefit from a stay. He is only seventeen years old, but has had rheumatism for two or three years, which has left his joints stiff (but not rigid), and we want to try this as a means to his complete recovery, and we have been told if he could go where he could get plenty of sunshine and an equal temperature he would have a better chance. I am an hospital nurse, and my three years' training is nearly finished, and I thought you might be able to tell me if I could get employment at any of those places, as I should like to be near him. Please also say what is the best time for going abroad.—*Merci.*

Your brother would not be wise to venture abroad except under the direction of a competent medical man who knows him; otherwise he may spend money and derive no benefit. Your best plan to obtain employment would be to seek an introduction to one of the resident doctors at the recommended health resort and to write to him, stating your circumstances and enclosing copies of one or two testimonials. When all preliminary questions are settled, apply to our "Travel" Editor for information as regards the place and journey.

*District Nursing.*

(76) Can you kindly tell me if it is safe for a nurse doing midwifery to attend cases of cancer? I have been nursing some years, and was always taught not to do so, it being considered dangerous to a maternity patient. I have been asked to do so on this district, so would be very glad of your advice on the subject.—*A. E. B.*

If you are nursing in a small place, and maternity cases are not frequent, no doubt you could attend to the cancer cases between times. But cancer cases when attended with discharge are generally extremely septic, and should not be nursed by anyone who is attending a confinement.

*The Outdoor Treatment of Consumption.*

(77) Is it true that there is a home established in the Mendip Hills, in Somersetshire, for the outdoor treatment of consumption?—*E. K., Brighton.*

Yes, there is such a home. The address is Nordrach-on-Mendip, Blagdon, and the principal is Dr. Thurnam.

## Figures and Tables

**Figure f1:**
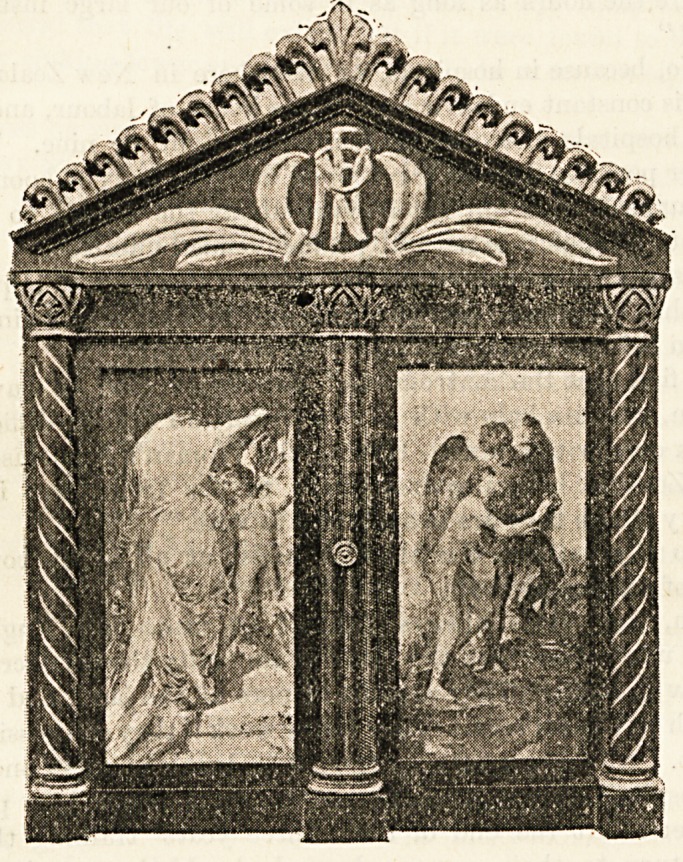


**Figure f2:**
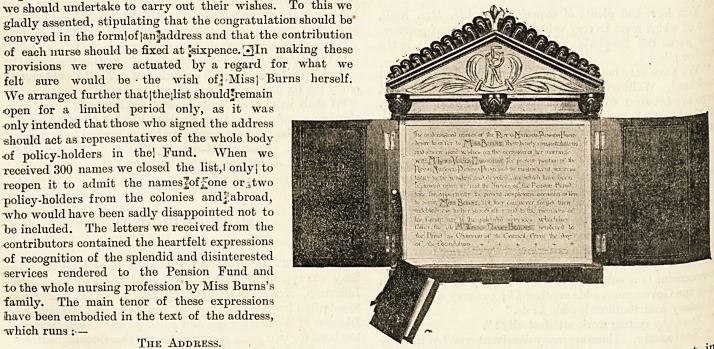


**Figure f3:**